# Supplementary material for: Tryptone in Growth Media Enhances Pseudomonas putida Biofilm
Source: Microorganisms. 2022 Mar 14;10(3):618. doi: 10.3390/microorganisms10030618 (PMC8954664; doi:10.3390/microorganisms10030618)
Supplement: Supplementary file 1 [file microorganisms-10-00618-s001.zip › microorganisms-1630936-supplementary.pdf]

Supplementary Materials

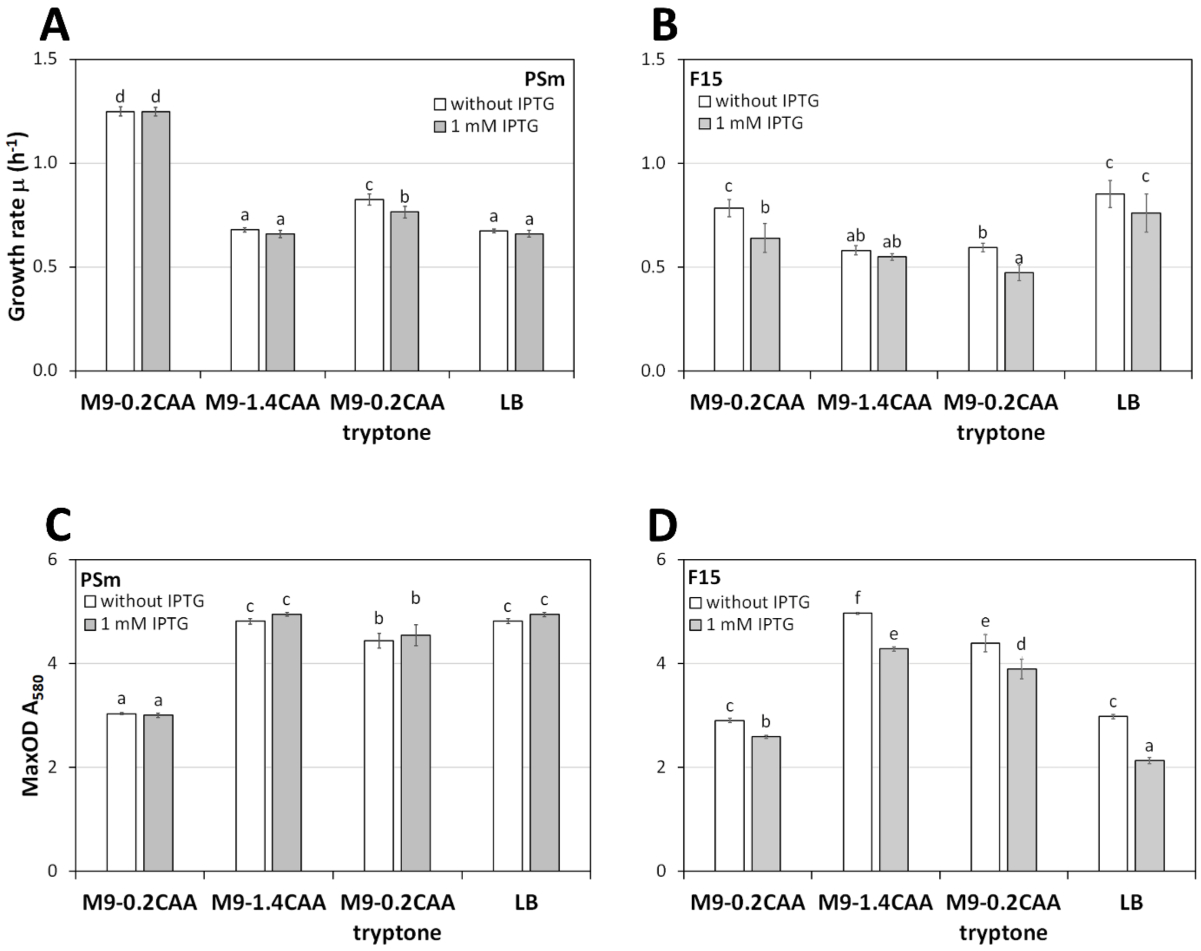

Figure S1. The growth parameters of planktic *P. putida* strains PSm and F15. (A, B) The growth rate and (C, D) maximum OD (maxOD) of *P. putida* grown in LB, M9-0.2CAA, M9-tryptone and M9-1.4CAA media. The arithmetical averages of at least three independent measurement sets are shown. Brackets show the 95% confidence intervals, and the homogenous groups are shown above columns by the lower-case letters. Identical letters denote non-significant differences ($p \geq 0.05$) between averages of biofilm.

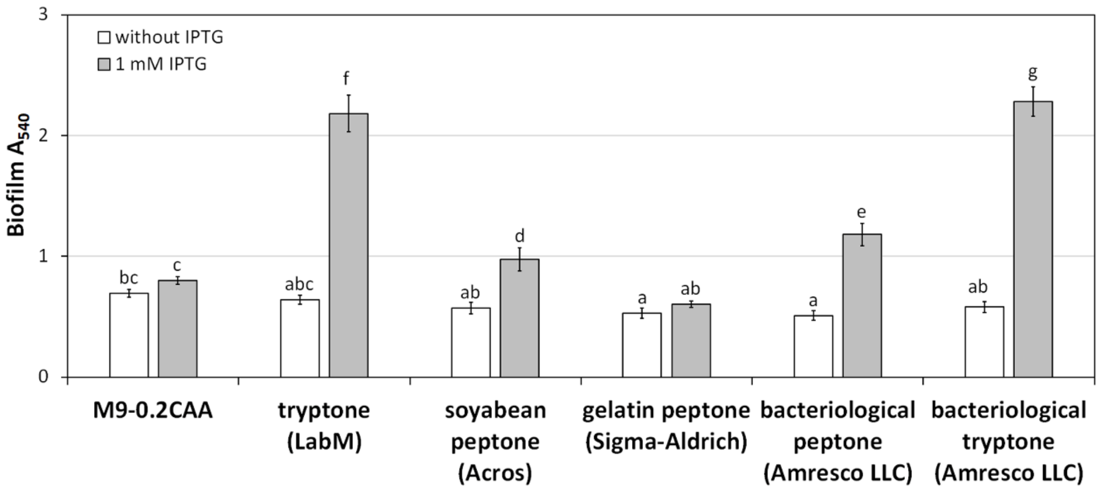

Figure S2. The biofilm of *P. putida* F15 in the M9-0.2CAA medium with a proteinaceous component of various origins. The arithmetical averages of at least three independent measurement sets are shown. Brackets show the 95% confidence intervals, and the homogenous groups are shown above columns by the lower-case letters. Identical letters denote non-significant differences ($p \geq 0.05$) between averages of biofilm.

.
